# Expression of Concern: An Efficient Hybrid Job Scheduling Optimization (EHJSO) approach to enhance resource search using Cuckoo and Grey Wolf Job Optimization for cloud environment

**DOI:** 10.1371/journal.pone.0357857

**Published:** 2026-09-08

**Authors:** The PLOS One Editors

After this article [1] was published, concerns were noted that the articles cited as References 32 and 33 were retracted before [1] was published.

The *PLOS One* Editors issue this Expression of Concern to inform readers of the concerns identified. Readers are advised to interpret the article [1] with caution.
