# Supplementary material for: An Efficient Hybrid Job Scheduling Optimization (EHJSO) approach to enhance resource search using Cuckoo and Grey Wolf Job Optimization for cloud environment
Source: PLoS One. 2023 Mar 13;18(3):e0282600. doi: 10.1371/journal.pone.0282600 (PMC10010551; doi:10.1371/journal.pone.0282600)
Supplement: Attachment — Submitted filename: Response to Reviewers.docx [file pone.0282600.s001.docx]

**Response to Reviewers**

| **Sl.No.** | **Comment** | **Response** |
| --- | --- | --- |
| **Reviewer :1** | | |
| 1 | The Abstract is somewhat long, so I suggest compressing the introduction of the research background. | Abstract initially had 289 words. It has been revised carefully without disturbing the objective of the paper and focused only the novelty and reduced to 245 words. |
| 2 | Job/workflow/task scheduling for cloud computing has attracted so much attention. The shortcomings of the existing works, such as https://ieeexplore.ieee.org/ document/8443134, should be further analyzed. | As suggested by the reviewer, we have surveyed the recommended the excellent paper and we got additional input to further refine our work. Thanks |
| 3 | As stated in the Abstract and Introduction, this manuscript does introduce a hybrid approach. But what are its new features? I suggest highlighting this aspect. | The main feature of this hybrid model is stated in the third bulletin point in page 3 and coloured red. |
| 4 | What is the allowable optimization delay when scheduling jobs in the public cloud? In some real-time application scenarios, the permissible delay is as low as milliseconds. But, the time overheads of cuckoo search and grey wolf techniques are considerable. It is necessary to investigate this respect. | Allowable optimization delay in the public cloud depends upon the communication between the edge device and the cloud server. As you rightly pointed that the optimization delay will be in milliseconds. In the manuscript Fig5 and table 2, the computation time analysis the time is measured in milliseconds. It is to be noticed that the computation time for the proposed work is less when compared to the existing works for various iteration. |
| **Reviewer 2** | | |
| 1 | Please simplify the abstract section by suggesting that the author focus more on the innovative points of the paper and their contributions. | Abstract is revised and simplified to 245 words. The main innovative idea is focused and is highlighted in the revised version of the manuscript. |
| 2 | Please update the serial number of the citation, e.g. in the first paragraph of the introduction, that is, “According to [28], cloud data centres will handle 94% of computing workload by 2021. ”. | It is wrongly referenced. Instead of [2] it has been mentioned as [28]. It is corrected and all the references are cross checked once again. |
| 3 | In the literature survey section, it is suggested that the author describe the relevant research results in recent years. I suggest the author to introduce some recent proposed meta-heuristics such as slime mould algorithm (SMA), Hunger Games Search (HGS), RUN Kutta optimizer (RUN), Colony Predation Algorithm (CPA) and Harris Hawk Optimization (HHO) to make the paper get more readers. | Thank you very much for your suggestion to do the literature on meta-heuristics algorithms. We have included all the suggested optimization algorithms and preferably cited available references from PLOS ONE journal in the literature survey section. Thank you very much for the valuable suggestions. |
| 4 | In the introduction section, the novelty of the work is missing. There is no sufficient justification as to why such a proposal is needed. It is recommended that the author elaborate on the shortcomings of the basic GWO and then elaborate on the main points about how your proposal will alleviate the existing problems of the GWO. | The introduction section is revised and the novelty of the work is refined and added in the objective at the end of introduction part. The same has been highlighted.  The performance of the proposed work is compared with four other similar works and the comparative analysis is discussed in section 4-Result and discussion |
| 5 | Please ask the author to standardize the abbreviations of the chart names. For example, it is suggested to change "Figure 1" to "Fig. 1". | All the chart names are changed to Fig. instead of Figure. |
| 6 | Please ask the author to standardize the description of formula symbols in the manuscript. For example, formula letters should be uniformly italicized, etc. | Common formatting as per the guidelines of PLOS ONE are followed through out the manuscript |
| 7 | The related work in section 2 is not clearly categorized. The related work section is too simplistic for this topic. To better highlight the related work, it is recommended that the author add a brief table of the main elements. | As per your suggestion, we have added a table with the references which have a major contribution to this work. |
| 8 | For the parameters α and β in Eq. (4), it is suggested that the author gives them in the text. Similarly, the parameters W1 and W2 in Eq. (20) are the same. | As per the suggestion of the reviewer, parameters α and β in Eq. (4) and W1 and W2 in Eq. (20) are given as text. |
| 9 | For Eq. (6), it is suggested that the author draws the levy distribution. | It is to be noticed that in Eq. (6) we mainly focus on the standard deviation of levy instead of the distribution. |
| 10 | Please ask the author to recheck the sentence, that is, “The contender has the best chance of succeeding the wolf as the new leader. acts as an advisor or consultant for”. | The sentence has been rechecked and reframed accordingly. |
| 11 | In between the concluding chapters, it is advisable to have a separate discussion section to analyze the experiments conducted in this paper in depth, what are the advantages and disadvantages compared to the existing methods? What are the specific shortcomings? To facilitate a better tour for the reader. | Section 4 is exclusively drafted for the analysis and discussion of the performance of the proposed EHJSOmethod. However as per the suggestion of the reviewer, we have added some more explanation at the end of section 4, which has been highlighted. |
| 12 | Limitations of this work should be added in the conclusion section. | Limitations of this work when we experiment has been added at the end of the conclusion. The future focus of this work also highlighted. |
| 13 | References should include DOI numbers, please reorganize and adjust them by the author. | DOI numbers for all the references have been added. |
